# Structure-Function Analysis of Porcine Cytochrome P450 3A29 in the Hydroxylation of T-2 Toxin as Revealed by Docking and Mutagenesis Studies

**DOI:** 10.1371/journal.pone.0106769

**Published:** 2014-09-03

**Authors:** Guyue Cheng, Changcun Liu, Xu Wang, Hongmin Ma, Yuanhu Pan, Lingli Huang, Haihong Hao, Menghong Dai, Zonghui Yuan

**Affiliations:** 1 MOA Laboratory for Risk Assessment of Quality and Safety of Livestock and Poultry Products, Huazhong Agricultural University, Wuhan, China; 2 National Reference Laboratory of Veterinary Drug Residues (HZAU), MOA Key Laboratory for Detection of Veterinary Drug Residues, Huazhong Agricultural University, Wuhan, China; 3 Key Laboratory of Combinatorial Biosynthesis and Drug Discovery (Ministry of Education) at the School of Pharmaceutical Sciences, Wuhan University, Wuhan, China; Cincinnati Childrens Hospital Medical Center, United States of America

## Abstract

T-2 toxin, one of the type A trichothecenes, presents a potential hazard to human and animal health. Our previous work demonstrated that porcine cytochrome P450 3A29 (CYP3A29) played an important role in the hydroxylation of T-2 toxin. To identify amino acids involved in this metabolic process, T-2 toxin was docked into a homology model of CYP3A29 based on a crystal structure of CYP3A4 using AutoDock 4.0. Nine residues of CYP3A29, Arg105, Arg106, Phe108, Ser119, Lys212, Phe213, Phe215, Arg372 and Glu374, which were found within 5 Å around T-2 toxin were subjected to site-directed mutagenesis. In the oxidation of nifedipine, the *CL*_int_ value of R106A was increased by nearly two-folds compared with the wild-type CYP3A29, while the substrate affinities and *CL*_int_ values of S119A and K212A were significantly reduced. In the hydroxylation of T-2 toxin, the generation of 3′-OH-T-2 by R105A, S119A and K212A was significantly less than that by the wild-type, whereas R106A slightly increased the generation of 3′-OH-T-2. These results were further confirmed by isothermal titration calorimetry analysis, suggesting that these four residues are important in the hydroxylation of T-2 toxin and Arg105 may be a specific recognition site for the toxin. Our study suggests a possible structure-function relationship of CYP3A29 in the hydroxylation of T-2 toxin, providing with new insights into the mechanism of CYP3A enzymes in the biotransformation of T-2 toxin.

## Introduction

T-2 toxin is a fungal sesquiterpenoid metabolite belonging to the type A trichothecenes, which are widely distributed in corns, oats and mixed feeds. Feeding with feeds contaminated by these toxins may lead to growth retard and production decline of livestock. Trichothecenes can also make animal sick and even cause death when the situation is serious, posing a potential threat to human health. The toxicity of trichothecenes is most likely a result of their ability to inhibit protein synthesis and partially inhibit RNA and DNA synthesis in cells [1].

T-2 toxin is metabolized in organisms to form various metabolites, resulting in detoxification of this toxin. HT-2 toxin, neosolaniol (NEO), 3′-OH-T-2, 3′-OH-HT-2, T-2 triol, T-2 tetraol, and some de-epoxy products are the common phase I metabolites of T-2 toxin [2], [3]. Biotransformation of T-2 toxin to HT-2 toxin in Vero cells and rat spleen lymphocytes resulted in a reduced inhibition of protein synthesis [4]. The two hydroxylated metabolites, 3′-OH-T-2 and 3′-OH-HT-2, were far less cytotoxic to *Reuber hepatoma* cells than the parent compound [5]. The decreasing order of the apoptosis-inducing activity of T-2 toxin and its metabolites was found to be T-2 toxin>3′-OH T-2 toxin> HT-2 toxin>3′-OH HT-2 toxin [6]. When T-2 toxin was biotransformed into NEO, there was a substantial decrease in the level of toxic activity [6], [7]. De-epoxy T-2 toxin was one-400th as toxic as T-2 toxin in a rat skin irritation bioassay [8]. Therefore, it is of vital significance to study the metabolism of T-2 toxin for the prevention of its potential hazard.

It is presently known that carboxylesterases are involved in the biotransformation of T-2 toxin into HT-2 toxin, cytochrome P450s take part in the 3′-hydroxylation of isovaleryl group of T-2 toxin, and epoxide hydrolases contribute most in the de-epoxy metabolism [9]. Previous studies demonstrated that cytochrome P450 3As (CYP3As) were involved in the hydroxylation of T-2 toxin in pigs. CYP3A22 and CYP3A46 mainly catalyzed T-2 toxin to generate 3′-OH T-2 [10], [11]. Recombinant porcine CYP3A29 was able to convert T-2 and HT-2 toxins into 3′-OH-T-2 and 3′-OH-HT-2, respectively [12]. Since CYP3A29 is highly expressed in the livers and small intestines of pigs and serves as the major CYP3A contributor [13], it can easily draw the conclusion that CYP3A29 contributes most in the hydroxylation of T-2 toxin among CYP3As in pigs. However, the catalytic mechanism of CYP3A29 metabolizing T-2 toxin is still unknown.

Cytochrome P450s are a superfamily of hemoproteins that play key roles in the metabolism of a wide variety of xenobiotic and endogenous compounds [14]. Although there is a wealth of information from X-ray crystal structures of mammalian P450s, the deep understanding about how these enzymes recognize a variety of structurally diverse chemicals is still limited. Among CYP3A subfamily, only crystal structures of human CYP3A4 have been resolved. The overall structure of CYP3A4 conforms to the fold that is characteristic of the P450 superfamily, with a C-terminal domain that contains the heme and the active site [15]. The crystal structures of CYP3A4 in complex with or without ligands demonstrate that the heme iron is ligated by a conserved cysteine (Cys442) and the propionates of the heme interact with the side chains of Arg105, Trp126, Arg130, Arg375, and Arg440 [16], [17]. Phe213 and Phe215 point toward the active site and form a “Phe-cluster” together with other five phenylalanines (Phe108, Phe219, Phe220, Phe241, and Phe304), which are involved in the initial recognition of the substrates [16].

Since P450 enzymes are highly dynamic and flexible, undergoing large conformational changes to allow substrate access and product release [18], it may not be necessary to study each enzyme-ligand interaction by crystallization due to the tedious procedure. The method of homology modeling and molecular docking in conjunction with site-directed mutagenesis has been extensively employed to study the substrate orientation within the active site of P450 enzyme and to identify potential residues involved in the positioning and catalytic mechanisms. Some results between crystallization or mutagenesis studies and modeling and docking studies were proven in satisfactory agreement [19]–[21].

In this study, in order to investigate the mechanism of porcine CYP3A29 in the hydroxylation of T-2 toxin, the homology model of CYP3A29 was constructed based on the crystal structure of ligand-free CYP3A4 (PDB ID: 1tqn) [17], which exhibits 76% amino acid sequence identity with CYP3A29 [22]. Molecular docking between CYP3A29 model and T-2 toxin molecule was performed to screen the possible amino acids responsible for the binding or catalyzing of T-2 toxin, followed by site-directed mutagenesis and enzymatic activity assay to investigate the functions of these residues in the hydroxylation of the toxin. It was demonstrated that Arg105 might be a specific recognition site of CYP3A29 for T-2 toxin. Besides, Arg106, Ser119 and Lys212 played important roles in the hydroxylation of T-2 toxin and the oxidation of nifedipine. This study sheds light on a possible structure-function relationship of porcine CYP3A29 in the hydroxylation of T-2 toxin, which may provide a deeper understanding for the metabolism of T-2 toxin in pigs.

## Materials and Methods

### Chemicals

Nifedipine (NIF), oxidized nifedipine (ONIF), glucose 6-phosphate, and anti-human CYP3A4 monoclonal antibody were purchased from Sigma (St. Louis, MO, USA). The goat anti-mouse IgG labeled with horseradish peroxidase (HRP) was from TIANGEN (Beijing, China). Immobilon Western HRP substrate was obtained from Millipore (Billerica, MA, USA). Bicinchoninic acid protein kit was purchased from Pierce (Rockford, IL, USA). The β-nicotinamide adenine dinucleotide phosphate sodium salt (β-NADP^+^) was from Roche (Beijing, China). T-2 toxin, Grace’s insect medium and fetal bovine serum were obtained from Invitrogen (Carlsbad, CA, USA). Insect GeneJuice Transfection Reagent was purchased from Merck (KgaA, Darmstadt, Germany). HPLC-grade methanol was purchased from Fisher Scientific (Waltham, MA, USA). All other chemicals and reagents commercially available were of the highest analytical grade.

### Cells and plasmids

*Spodoptera frugiperda* (Sf9) cells and baculovirus transfer vector pFastBac HTb were obtained from BD Gentest (Franklin Lakes, New Jersey, USA). *Esherichia coli* DH 5α, *E. coli* DH10Bac and pMD 18-T vector were purchased from Takara (Dalian, China). The plasmids pFastBac1-CYP3A29, pFastBac1-pNPR and pFastBac1-pb5 were previously constructed and stored in our lab [22].

### Homology modeling and molecular docking

Amino acid sequence alignment was performed with Clustal X [23]. The amino acid sequence of CYP3A29 was screened against the PDB structure database (http://www.rcsb.org/pdb/home/home.do). From a series of templates, the crystal structure of ligand-free CYP3A4 (PDB ID: 1tqn) [17], exhibiting the highest sequence identity of 76% with CYP3A29, was selected as the modeling template. A three dimensional model of CYP3A29 was constructed by homology modeling tool Modeller [24]. The model with the highest score was validated by Procheck [25], which evaluated the stereochemical quality of a protein structure by analyzing residue-by-residue geometry and overall structural geometry.

The structure model of T-2 toxin, whose energy was minimized using the molecular mechanics method (MM2), was generated by ChemDraw (CambridgeSoft, Waltham, MA, USA). The docking on the monomer model of CYP3A29 with T-2 toxin was performed using Autodock 4.0 [26], which is based on Lamarckian Genetic Algorithm (LGA), a hybrid genetic algorithm with local optimization that uses a parameterized free-energy scoring to estimate the binding energy. In this docking simulation, semi-flexible docking protocols were used, in which the target protein CYP3A29 was kept as rigid. The Kollman charges, solvation parameters and polar hydrogen were added into the water free CYP3A29 model for the preparation of protein in docking simulation. The substrate T-2 toxin was treated as flexible ligand by modifying their rotatable torsions. The rigid root of the T-2 toxin ligand was defined automatically. All rotatable dihedrals in the ligand were assigned by Auto-Tors program and were allowed to rotate freely. AutoGrid 4.0 program was employed for generating grid maps for the ligands. The grid box was fixed in the catalytic active region between heme moiety and Ser 119 of CYP3A29. The box size was set as 60 Å×60 Å×60 Å to cover the whole active site and let the ligand rotate freely. The spacing between grid points was 0.375 Å. The LGA was utilized to search for the best conformer of the ligand. The individual LGA executions were clustered and ranked to generate the final docking model, and the potential binding sites were defined as amino acids in distances of less than 5 Å to T-2 toxin.

### Site-directed mutagenesis

Using plasmid pFastBac1-CYP3A29 [22] as template, the mutants were constructed by one step site-directed mutagenesis [27] by using the primers shown in Table 1. The constructed plasmids were sequenced by GenScript (Nanjing, China) and confirmed to be correct.

**Table 1:** Primers for site-directed mutagenesis.

Primer	Sequence (from 5′ to 3′ end)
R105A	Fwd: CTGTCTTCACAAAC***GCG***AGGTCTTTTGGTCC
	Rev: GGACCAAAAGACCT***CGC***GTTTGTGAAGACAG
R106A	Fwd: TATTCTGTCTTCACAAACCGG***GCG***TCTTTTGGTCCATTG
	Rev: CAATGGACCAAAAGA***CGC***CCGGTTTGTGAAGACAGAATA
F108P	Fwd: CTTCACAAACCGGAGGTCT***GCT***GGTCCATTGGGCGCTA
	Rev: TAGCGCCCAATGGACC***AGC***AGACCTCCGGTTTGTGAAG
S119A	Fwd: TATGAGAAACGCTCTC***GCT***CTGGCTGAGGATGAAG
	Rev: CTTCATCCTCAGCCAG***AGC***GAGAGCGTTTCTCATA
K212A	Fwd: CCCCTTTGTGGAAAACAGCAAGAAGCTCTTA***GCC***TTTAGTTTCTTTG
	Rev: CAAAGAAACTAAA***GGC***TAAGAGCTTCTTGCTGTTTTCCACAAAGGGG
F213A	Fwd: CCCCTTTGTGGAAAACAGCAAGAAGCTCTTAAAA***GCG***AGTTTCTTTG
	Rev: CAAAGAAACT***CGC***TTTTAAGAGCTTCTTGCTGTTTTCCACAAAGGGG
F215A	Fwd: GCAAGAAGCTCTTAAAATTTAGT***GCC***TTTGATCCATTCCTTCTCTC
	Rev: GAGAGAAGGAATGGATCAAA***GGC***ACTAAATTTTAAGAGCTTCTTGC
R372A	Fwd: CCCAATTGCTGCT***GCA***CTTGAGAGGGCCTGTAAG
	Rev: CTTACAGGCCCTCTCAAG***TGC***AGCAGCAATTGGG
E374A	Fwd: GCTAGACTT***GCG***AGGGCCTGTAAGAAGGATG
	Rev: CATCCTTCTTACAGGCCCT***CGC***AAGTCTAGC

Note: The mutated sites are underlined in bold and italic.

### Expression of CYP3A29 and its mutants

The empty vector, pFastBac HTb, and the recombinant vectors, pFastBac1-CYP3A29 [22] (wild-type) and its variants, were transformed into *E. coli* DH10Bac cells, respectively. The positive clones were selected as white color, using 5-bromo-4-chloro-3-indolyl-β-D-galactopyranoside (X-gal) as the substrate. The recombinant baculovirus DNA (Bacmid-CYP3A29 or its mutants) was isolated from *E. coli* DH10Bac positive clones and then transfected into Sf9 cells using Insect GeneJuice Transfection Reagent. The medium containing viruses was collected three to four days post transfection and stored as virus stock.

For each batch of enzyme preparation, Sf9 cells were cultured to 2×10^7^ cells/liter in Grace’s insect medium, and the cells were infected with recombinant viruses at six times higher concentration than that under expression conditions. The cells were co-infected with viruses encoding pNPR (porcine NADPH-P450 reductase) and pb5 (porcine cytochrome b5) as described previously [22]. The Sf9 cell lines were maintained in flasks (75 cm^2^) at 27°C in Grace’s medium containing 10% fetal bovine serum and 6 µM heme complexes of hemoglobin. The Sf9 cells were harvest after culturing for 72 h, and the microsomes were prepared by ultrasonication and differential speed centrifugation (10,000 and 105,000 *g*).

The microsomal protein content was determined according to Lowry et al. [28] using the bicinchoninic acid protein assay (Pierce). The content of P450 was measured using the carbon monoxide-difference spectrum [29], the co-expressed pNPR activity was measured using the cytochrome *c* reduction assay [30], and the concentration of co-expressed pb5 was estimated according to the method described previously [31]. After quantification, the microsomal proteins were frozen and stored at −70°C until use.

### SDS-PAGE and immunoblot analysis

The SDS-PAGE was carried out with glycine-Tris buffer system [32]. The gel was stained with Coomassie brilliant blue G-250. For immunoblot analysis, microsomal proteins separated by SDS-PAGE were electro-transferred to a polyvinylidene difluoride membrane. The blots were developed with the primary antibody raised against human CYP3A4 (MAb HL3, dilution 1∶1000) followed by the goat anti-mouse IgG labeled with HRP (dilution 1∶20000). The individual blots were visualized on X-film using luminol chemiluminescent reagent of Immobilon Western HRP substrate.

### Assay for enzymatic activity of CYP3A29 and its mutants

NIF oxidation activity was measured as previously described [22]. The reaction mixture (200 µL) for the assay consisted of NIF (0∼80 µM) as substrate, recombinant CYP3A29 or its mutants (5 pmol of P450), and the NADPH-generating system (2 mM NADP, 20 mM glucose 6-phosphate, 2 U/mL glucose 6-phosphate dehydrogenase and 5 mM MgCl_2_) in 50 mM potassium phosphate buffer (pH 7.4). All incubations were conducted in triplicate. The reactions were initiated by addition of the NADPH-generating system after preincubation at 37°C for 5 min. After incubation at 37°C for 10 min, the reaction was terminated by addition of 50 µL of ice-cold 15% (w/v) trichloroacetic acid, followed by centrifugation for 20 min at 10,000 *g* to precipitate the proteins. The supernatants were subjected to HPLC analysis.

The HPLC condition was set as previously described [22]. The formation of ONIF in the reaction mixture was determined based on calibration curves constructed from a series of standards of 0.01∼1 µM ONIF. Intraday (*n* = 5) and interday (*n* = 5) precisions did not exceed 10% in any of the assays. Kinetic parameters for the oxidation of NIF were calculated by fitting the data to the Hill equations, v = *V*_max_ [S]*^n^*/(*K*_m_+[S]*^n^*), using nonlinear regression analysis. Equations were selected by goodness of fit based on R^2^ values and least residual sum of squares. The intrinsic clearance (*CL*_int_) was considered as the ratio of *V*_max_ to *K*_m_.

### Identification and semi-quantitation of T-2 toxin metabolites

The reaction mixture (200 µL) for the assay consisted of 40 µM of T-2 toxin as substrate, recombinant CYP3A29 or its mutants (10 pmol of P450), and the NADPH-generating system cited above in 50 mM Tris-HCl buffer (pH 7.4). All incubations were conducted in triplicate. The reaction mixture was initiated by the addition of the NADPH-generating system. The reaction mixture was maintained at 37°C for 90 min, and the reaction was terminated by addition of 50 µL of ice-cold 15% (w/v) trichloroacetic acid. Equal volume of ice-cold acetonitrile (100 µL) was added to terminate the reaction. The mixture was vortexed and centrifuged at 15,000 *g* for 15 min. The supernatant was filtered through a 0.22 µm microbore cellulose membrane, and 10 µL of aliquot was analyzed by ESI-IT-TOF mass spectrometry coupled with a high-performance liquid chromatography system (LC/MS-IT-TOF, Shimadzu, Kyoto, Japan) according to Wu et al [12] to identify the metabolites. The peak area of hydroxylation products was used for semi-quantitative analysis.

### Isothermal titration calorimetry (ITC) measurement

Calorimetry pool was filled with CYP3A29 or its mutants (10 pmol of P450) and 1 mM NADPH, and the pool was thermostated to 37°C and stirred at 502 rpm. After the calorimeter was balanced for 60 s, 100 µM T-2 toxin was titrated by single drop. Then the toxin was continuously titrated by 20 drops, each drop for 10 µL with an interval of 300 s, and the heat and the time were recorded.

The ITC experiment directly measured the thermal power (P). Each peak represented the thermal effect associated with each injection. The heat evolved (Q) from each injection was calculated according to the following formula: P = dQ/dt. The Q generated by the enzymatic conversion of substrate to product was calculated as the difference between Q of the enzyme sample and Q of the dilution buffer.

### Statistical analyses

Descriptive statistical parameters such as mean and standard deviation were calculated using Microsoft Excel 2003. Statistical analyses were performed with Student’s t-test and Bonferroni revision, and differences were considered to be statistically significant when the *p*-value was <0.05 or <0.01.

## Results

### Homology model of porcine CYP3A29 and docking of T-2 toxin

Amino acid sequence alignment demonstrated that the second structures and the substrate recognition sites of porcine CYP3A29 (GenBank ID: Z93099), CYP3A22 (GenBank ID: AB006010), CYP3A46 (GenBank ID: EF625347) and human CYP3A4 (GenBank ID: AF182273) were substantially identical (Fig. S1). The X-ray crystal structure of CYP3A4 (PDB ID: 1tqn) was chosen as template since it exhibited highest protein sequence identity (76%, 382 aa/503 aa) to that of CYP3A29. Among the five models generated by the MODELER program, the model with the lowest probability density function (PDF) total energy and discrete optimized potential energy (DOPE) score was selected and further refined by energy minimization. The quality of the refined model was assessed by PROCHECK Ramachandran plot, demonstrating that 91.5% of the residues were in the most favored regions, 6.9% in additional allowed regions, and 0.5% in generously allowed regions. Overall, the refined model of CYP3A29 was all well within the acceptable range with high quality.

**Figure 1:**
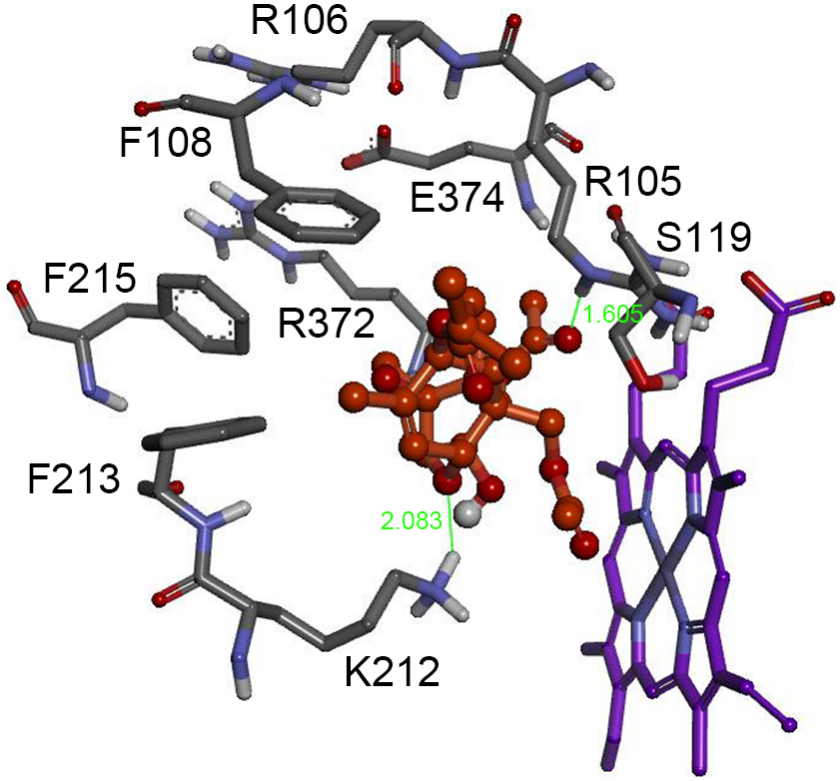
The binding pocket of porcine CYP3A29 docked with T-2 toxin. CYP3A29 interaction residues, Arg105, Arg106, Phe108, Ser119, Lys212, Phe213, Phe215, Arg372 and Glu374, are in distances within 5Å to T-2 toxin molecule. The T-2 toxin is colored in orange. The heme is represented in violet color. The green lines and numbers denote hydrogen bonds and bond lengths.

When T-2 toxin was docked into CYP3A29, nine residues, Arg105, Arg106, Phe108, Ser119, Lys212, Phe213, Phe215, Arg372 and Glu374, were found to be located in a distance within 5Å to T-2 toxin, among which, Arg105 and Lys212 formed hydrogen bonds with the toxin molecule (Fig. 1).

### Heterologous expression of CYP3A29 and its variants in Sf9 insect cells

The screened nine amino acids, including 4 basic residues (Arg105, Arg106, Lys212 and Arg372), 1 acidic residue (Glu374), 1 polar residue (Ser119), and 3 phenylalanines (Phe108, Phe213 and Phe215) were respectively replaced with the small non-polar residue, alanine. Porcine CYP3A29 or its mutants were co-expressed with pNPR and pb5 in Sf9 insect cells, and about 50 pmol of P450, 200 units of pNPR and 110 pmol of pb5 were detected per 10^6^ cells. The expression of each CYP3A29 variant, as presented in the microsomal proteins, was detected by immunoblot analysis using anti-CYP3A4 antibodies (Fig. S2), and the molecular mass of each mutant was about 55 kDa, in consistence with the theoretical molecular mass of CYP3A29 of 57188.7 Da.

### Kinetic analysis of CYP3A29 and its mutants in the oxidation of nifedipine

**Figure 2:**
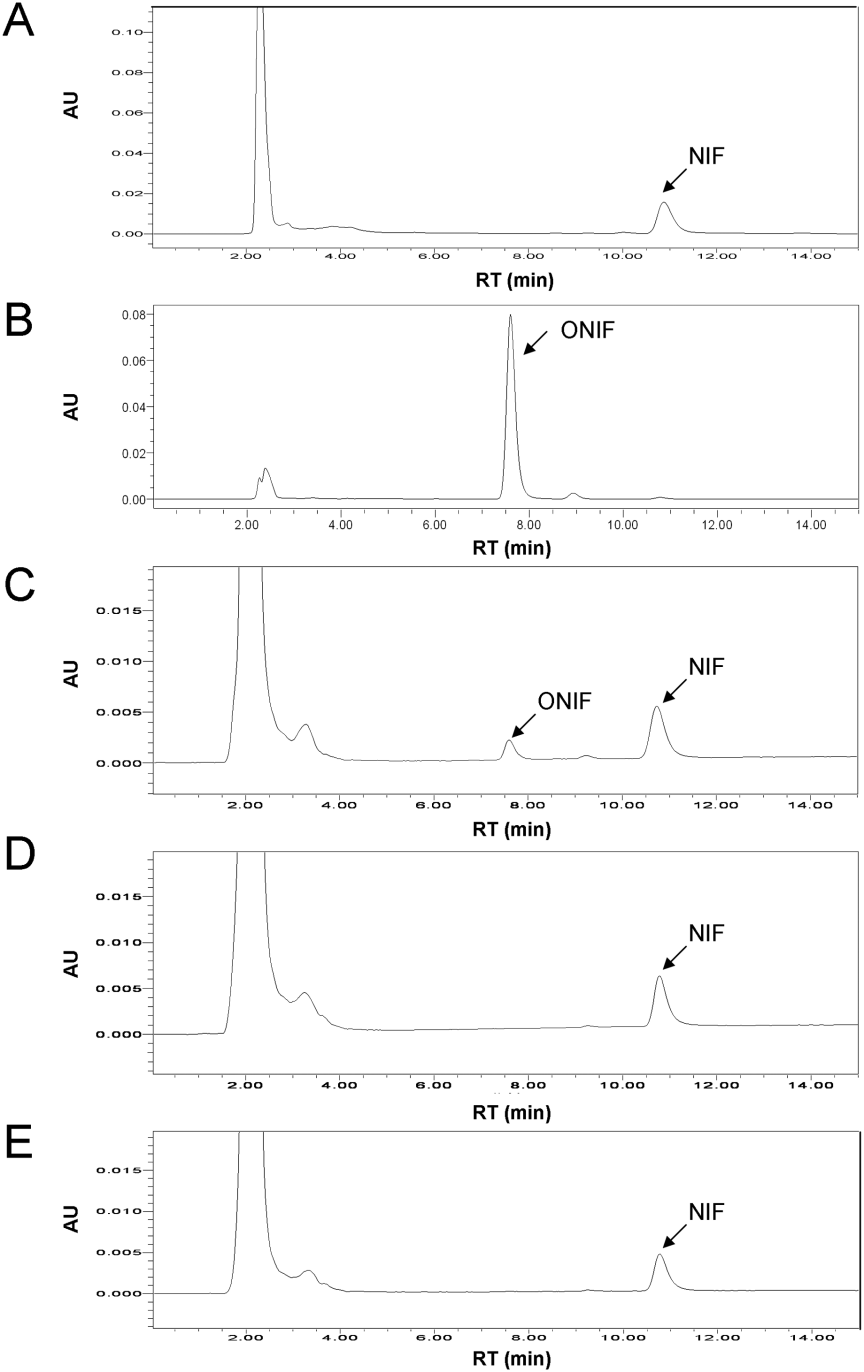
HPLC chromatograms of the metabolite of nifedipine (NIF) catalyzed by recombinant CYP3A29. (A) NIF standard. (B) Oxidized nifedipine (ONIF) standard. (C) NIF incubated with Sf9 microsomes containing the recombinant CYP3A29 at 37°C for 30 min in the presence of NADPH-generating system. (D) NIF incubated with Sf9 microsomes free of the recombinant CYP3A29 at 37°C for 30 min in the presence of NADPH-generating system. (E) NIF incubated with Sf9 microsomes containing the recombinant CYP3A29 at 37°C for 30 min in the absence of NADPH-generating system.

As shown in Fig. 2, when NIF was incubated with Sf9 microsomes containing the recombinant CYP3A29, the oxidized product, ONIF, was generated (Fig. 2C), while no ONIF was generated when NIF was incubated with the microsomes of Sf9 cells transfected with the empty vector (Fig. 2D) or with microsomes containing the recombinant CYP3A29 but in the absence of NADPH-generating system (Fig. 2E), demonstrating that the heterogeneously expressed CYP3A29 was of biological activity. Equal amount (5 pmol of P450 in 200 µL reaction mixture) of each CYP3A29 variant was used for the subsequent enzymatic activity assays.

The kinetic data for the generation of ONIF by wild-type and each variant CYP3A29 were examined. The relationship between substrate concentration and reaction velocity is shown in Fig. S3, and the *V*_max_ and *K*_m_ values are listed in Table 2. The *V*_max_/*K*_m_ values, representing intrinsic clearance (*CL*_int_), were calculated and compared between wild-type CYP3A29 (*V*_max_, 8.53±0.69 nmol/min/nmol P450; *K*_m_, 15.68±2.96 µmol/L; *CL*_int_, 0.54±0.03 mL/nmol P450/min) and each mutant. The *V*_max_ of S119A was slightly increased (11.03±0.85 nmol/min/nmol P450) compared with the wild-type, but the *K*_m_ value (24.15±3.68 µmol/L) was predominantly increased by nearly 1.6 folds, resulting in a weak decrease of *CL*_int_ value (0.45±0.01 mL/nmol P450/min). Similarly, K212A also exhibited a higher *K*_m_ (21.71±1.83 µmol/L) and a lower *CL*_int_ (0.46±0.03 mL/nmol P450/min). In contrast, the NIF oxidation activity of CYP3A29 was significantly increased by replacing Arg106 with Ala. R106A exhibited a slight increase in *V*_max_ (9.86±0.37 nmol/min/nmol P450), a notable decrease in *K*_m_ (10.63±0.93 µmol/L), and accordingly the *CL*_int_ (0.93±0.02 mL/nmol P450/min) was nearly twice as much as that of the wild-type. No statistically significant differences were identified in other mutants.

**Table 2:** Enzyme kinetic parameters of recombinant CYP3A29 and its mutants oxidizing nifedipine.

Enzymes	SRS	*V*_max_ (nmol/min/nmol P450)	*K*_m_ (µmol/L)	*CL*_int_ (mL/nmol P450/min)	*n*
Wild-type		8.53±0.69	15.68±2.96	0.54±0.03	1.10
R105A	SRS1	8.42±0.71	14.60±3.07	0.58±0.07	1.09
R106A	SRS1	9.86±0.37*	10.63±0.93*	0.93±0.02**	1.34
F108A	SRS1	7.54±0.57	13.43±2.37	0.56±0.03	1.26
S119A	SRS1	11.03±0.85*	24.15±3.68*	0.45±0.01**	1.41
K212A		10.02±0.89	21.71±1.83*	0.46±0.03*	1.48
F213A		8.45±0.61	14.50±2.45	0.58±0.04	1.25
F215A		9.46±1.03	17.67±4.45	0.53±0.08	1.18
R372A	SRS5	8.58±0.78	15.88±3.60	0.54±0.05	1.07
E374A	SRS5	8.48±0.72	17.05±3.08	0.50±0.05	1.41

Note: The values are expressed as means ± standard deviations of the results of three independent experiments. *n* indicates the Hill coefficients.

**(*p*<0.01) and *(*p*<0.05) indicate statistically significant difference between the wild-type and the mutant.

### Metabolizing of T-2 toxin by CYP3A29 and its mutants

**Figure 3:**
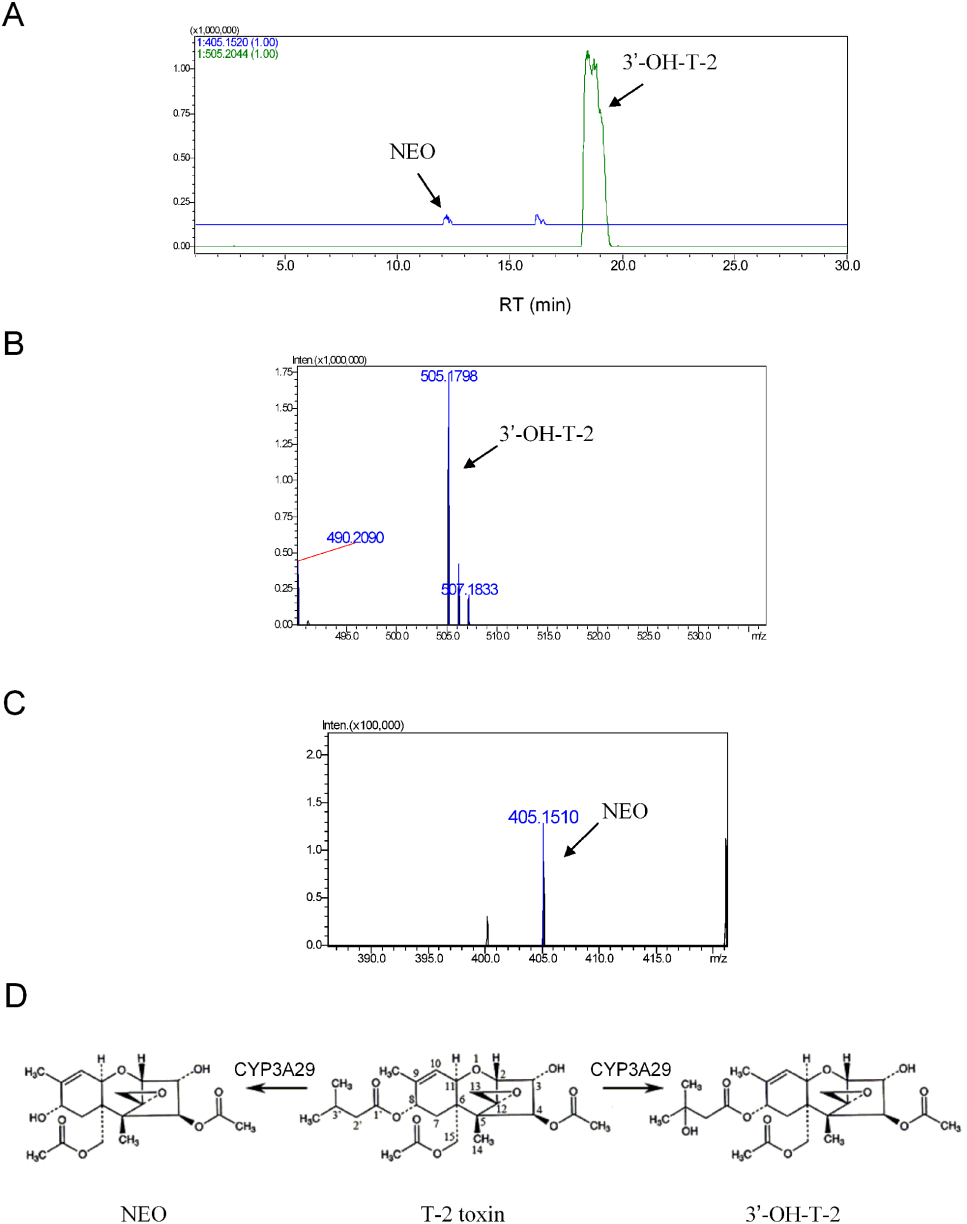
Identification of the metabolites of T-2 toxin after incubation with recombinant CYP3A29. (A) Accurate extracted ion chromatograms of the metabolites of T-2 toxin after incubation with CYP3A29. (B) Accurate MS spectrum of 3′-OH-T-2. (C) Accurate MS spectrum of NEO. (D) Structural illustration of the metabolites of T-2 toxin metabolized by CYP3A29.

Previous study demonstrated that 3′-OH-T-2 and NEO were detected as the metabolites of T-2 toxin when incubating with recombinant pig CYP3A29 [12]. Similarly, T-2 toxin was shown in this study to be metabolized to 3′-OH-T-2 and NEO by the Sf9 microsomes containing recombinant CYP3A29 (Fig. 3) or its mutants (Fig. S4), while nearly no 3′-OH-T-2 or NEO was found by the microsomes containing no expressed recombinant CYP3A29 (vector control). The relative amount of 3′-OH-T-2 was much higher than that of NEO (Fig. 3 and Fig. S4), suggesting that the hydroxylated metabolite was the major metabolite.

**Figure 4:**
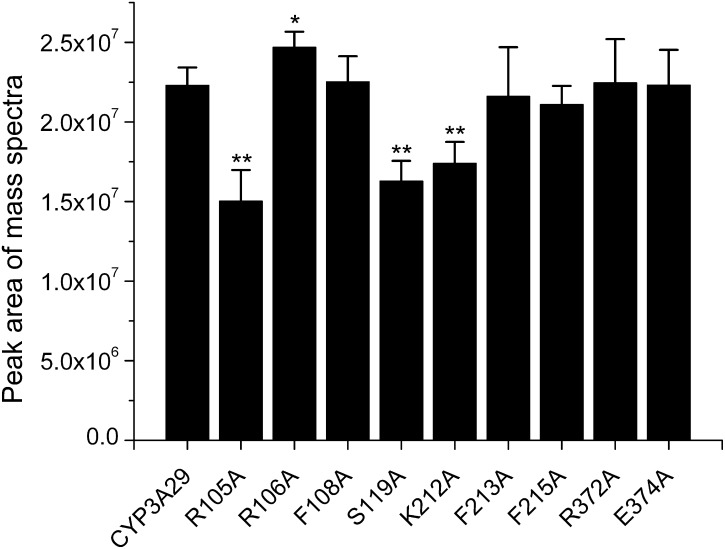
The peak area of the mass spectra of 3′-OH-T-2 generated by CYP3A29 metabolizing T-2 toxin. The data were expressed as means ± standard deviations (error bars) (n = 3). **(*p*<0.01) and *(*p*<0.05) indicate statistically significant difference between the wild-type CYP3A29 and each mutant.

Unfortunately, there was no 3′-OH-T-2 standard compound and the yield of 3′-OH-T-2 could not be calculated by the substrate disappearance due to that the biotransformation of T-2 toxin by CYP3A29 did not result in only one metabolite (i.e. 3′-OH-T-2). Therefore, the production of 3′-OH-T-2 by CYP3A29 or its mutants was semi-quantitated and compared according to the peak areas of the LC/MS data. As shown in Fig. 4, the yields of 3′-OH-T-2 by R105A (1.48-fold change), S119A (1.37-fold change) and K212A (1.28-fold change) were decreased significantly, while was slightly increased by R106A (1.11-fold change), compared with the wild-type. The hydroxylation of T-2 toxin by other mutants had no significant changes.

**Figure 5:**
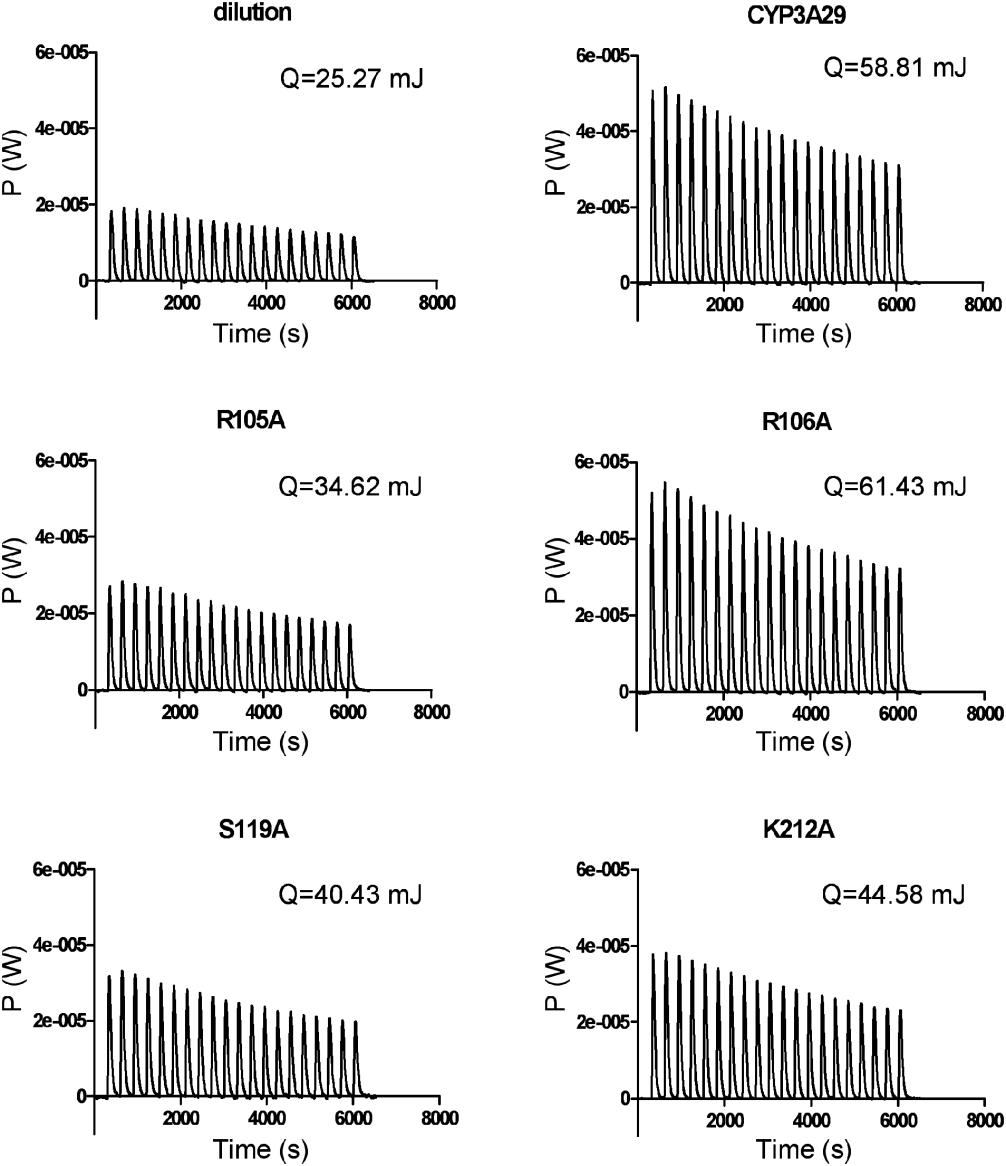
The thermogram of CYP3A29 and its mutants metabolizing T-2 toxin.

The above four mutants affecting the metabolism of T-2 toxin were further confirmed by ITC measurement. As shown in Fig. 5, the thermal powers of R105A, S119A and K212A in the metabolism of T-2 toxin were significantly reduced compared with the wild-type, while the thermal power of R106A was increased slightly. Heat evolved (Q_enzyme_-Q_dilution_) from the metabolism of T-2 toxin by R105A, S119A and K212A also changed significantly. The heats evolved by R105A (9.35 mJ, 3.59-fold change), S119A (15.18 mJ, 2.21-fold change) and K212A (19.31 mJ, 1.74-fold change) were decreased significantly compared with the wild-type (33.54 mJ). The heat evolved by R106A (36.16 mJ, 1.08-fold change) was slightly increased compared with that of the wild-type. Since the ITC measured the total heat produced by the enzymatic reaction, the ITC data manifested the biotransformation of T-2 toxin to both 3′-OH-T-2 and NEO. The NEO formation (hydrolysis of the C8-isovaleryloxy ester of T-2 toxin) was a heat absorbed reaction but not a major reaction catalyzed by CYP3A29, thus the fold changes of these four residues were different between the ITC data and the LC/MS data but with a similar tendency.

## Discussion

This study demonstrates that porcine CYP3A29 is able to metabolize T-2 toxin to form 3′-OH-T-2 and NEO, implying that CYP3A29 is involved in the hydroxylation of the isovaleryl group and the hydrolysis of the C8-isovaleryloxy ester of T-2 toxin. These two biotransformation processes make T-2 toxin more polar and water-soluble to exclude from the body. Hydroxylation and hydrolysis metabolism of T-2 toxin are thought to reduce the toxic effects of the toxin [5], [33], therefore, CYP3A29 is very important in the detoxification of T-2 toxin. Similarly, previous studies demonstrated that T-2 toxin could be metabolized to 3′-OH-T-2 by porcine CYP3A22 and CYP3A46 [10], [11], but those studies did not test the carboxylesterase activity (hydrolysis of the ester bond) of porcine CYP3As for T-2 toxin. It is reported that human CYP3A4 can mediate the hydrolysis of the ester bond [34], [35], inferring that human CYP3A4 and porcine CYP3A29 are orthologous proteins. Based on the metabolic study of T-2 toxin in pigs, the metabolic process of T-2 toxin in the human body can be speculated. Nevertheless, the hydroxylation of T-2 toxin was the main reaction catalyzed by CYP3A29, and the hydrolysis reaction was quite weak. Conclusively, porcine CYP3As play a vital role in the hydroxylation of T-2 toxin in pigs.

Although the homology model of porcine CYP3A29 is very similar to the crystal structure of human CYP3A4, CYP3A29 has a relatively lower NIF oxidase activity than CYP3A4 [22]. The most distinct differences in the amino acid sequences of these two enzymes were found to be located in the helices F–G region (Fig. S1), and this region was considered important in determination of the substrate specificity of CYP3As [36]. In CYP3A4, Leu210 is implicated in the effector binding as well as the stereo- and regio-selectivity profile and both Leu211 and Asp214 are implicated in the cooperativity of the enzyme [37], [38]. Furthermore, a variety of studies have concentrated upon the ability of CYP3A4 to accommodate more than one substrate molecule simultaneously and the resultant activation/enhancer effects [38]–[41]. It is also reported that CYP3A29 displays ligand binding that does not follow the Michaelis-Menten kinetics, but in the sigmoidal or autoactivation kinetic behaviors with Hill coefficients (*n*) >1 [22]. Sigmoidal kinetics can be interpreted by the allosteric effect hypothesis. CYP3As usually undergo dramatic conformational changes upon ligand binding with an increasing volume in the active site, which could enhance the adaptability of enzyme to the ligands [42]. In this study, the mutants of CYP3A29 displayed different Hill coefficients in the oxidation of NIF, but all with *n>*1 (Table 2 and Fig. S3). The Hill coefficients of R106A (*n* = 1.34), S119A (*n* = 1.41), K212A (*n* = 1.48) and E374A (*n* = 1.41) were increased significantly compared with the wild-type (*n* = 1.10), indicating that these mutations enlarged the volume of the active site.

In the metabolism of NIF, among the amino acids determined by docking T-2 toxin into CYP3A29, R106A exhibited an increased *V_max_* and a reduced *K_m_*, resulting in that the metabolic capacity of R106A improved significantly by nearly two-fold compared with that of the wild-type. Arg106 is located in SRS1 (SRS, substrate recognition site), when it was mutated into the small residue, alanine, the steric hinderance was reduced as well as the charge was eliminated, thus NIF might be more easily to get into the active center. In contrast, S119A substitution weakened the ability of CYP3A29 metabolizing NIF, with higher *K*_m_ thus lower *CL*_int_. Ser119 is conservative among CYP3A subfamily members. Studies show that Ser119 of CYP3A4 has a major impact on the substrate stereoselectivity [43], [44], therefore, S119A substitution may affect the orientation of NIF in the active center of the enzyme, thus affect its oxidation. Similarly, K212A also exhibited a higher *K_m_* and a lower *CL*_int_. Helices F-G are important for the substrate specificity of CYP3As [36]. This region is recognized to adopt different conformations dependent on the presence of different ligands. It was shown that broad substrate specificity of CYP3A4 stemed from the malleability of the F–F’ loop (residues 211–218, between helix F and F’) that resided in the vicinity of the channel connecting the active site and bulk solvent [45]. Arg212, located in this loop, was found in the active site in a ligand-free structure of CYP3A4, but moved towards the surface of the enzyme in the ketoconazole-CYP3A4 complex [42]. Therefore, K212A substitution might affect the transportation of NIF to the active center of CYP3A29.

In the hydroxylation of T-2 toxin by CYP3A29, since there was no standard compound of 3′-OH-T-2, only semi-quantitative assay was done by using LC/MS. R105A, S119A and K212A substitutions significantly reduced the generation of the hydroxylation product, 3′-OH-T-2, while R106A substitution slightly increased the hydroxylation rate (Fig. 4). This result was further confirmed by the isothermal titration calorimeter analysis based on the heat evolved produced by CYP3A29 and its mutants (Fig. 5). According to the results of molecular docking, T-2 toxin form hydrogen bonds with Arg105 and Lys212 in CYP3A29. Hydrogen bonding, electrostatic interaction, and π-π-conjugated contribute to the orientation of the substrate in the active center [46]–[48]. Mutation at these two sites might destroy the formation of hydrogen bonds between T-2 toxin and CYP3A29, influencing the orientation of the toxin molecule in the active-site cavity of the enzyme, thus the yields of 3′-OH-T-2 by R105A or K212A were much lower than that by the wild-type. In addition, S119A also exhibited a weaker metabolic capacity in the hydroxylation of T-2 toxin, and Ser119 might affect the orientation of T-2 toxin in the active center as in the case of NIF oxidation. Compared with the wild-type, the catalytic capability of R106A in the metabolism of NIF was increased more obviously than in the metabolism of T-2 toxin (Table 2 and Fig. 4). This may be due to the different kinetic characteristics of the enzyme for these two different substrates. The oxidation of NIF by CYP3A29 followed an auto-activation kinetic behavior as discussed above and R106A substitution might make the substrate access the active center more easily, while the metabolism of T-2 toxin by CYP3A29 was likely to be a hyperbolic saturation kinetics process. Combining the results of CYP3A29 metabolizing NIF and T-2 toxin, it can be speculated that Arg105, Arg106, Ser119 and Lys212 of CYP3A29 are crucial to the substrate positioning and binding. Furthermore, since R105A substitution did not influence the oxidation of NIF, Arg105 might be a unique recognition site of CYP3A29 for T-2 toxin.

Other sites, including Phe108, Phe213, Phe215, Arg372 and Glu374 did not show significant impact on the metabolism of NIF or T-2 toxin. Previous studies showed that Phe108, Phe213 and Phe215 formed the “Phe-cluster” on the ceiling of the active-site cavity of CYP3A4 [15]. When binding large substrates, phenylalanine residues in the “Phe-cluster” were repositioned, resulting in an extension of helix F and a larger active site [16], [42]. However, F108A, F213A and F215A substitutions did not change the metabolic capacity for either NIF or T-2 toxin in our study. Correspondingly, the Hill coefficients of these mutants didn’t alter much when compared with that of the wild-type (Table 2). This may be due to the small molecular sizes of the NIF (*M*_r_ 346.34) and T-2 toxin (*M*_r_ 466.53), which might not cause great conformation changes in the active center of CYP3A29, thus mutation of “Phe-cluster” did not influence the metabolism of these two substrates. This is in consistence with the case of progesterone (*M*_r_ 314.47), which induced very little conformational change in CYP3A4 [16]. R372A and E374A, although located in SRS5, did not have significant impact on the metabolic capability either, suggesting they are not the functional sites of CYP3A29 for metabolizing NIF or T-2 toxin.

In conclusion, our study revealed that Arg105, Arg106, Ser119, Lys212 might be important to the function of CYP3A29 in the metabolism of NIF or T-2 toxin, and Arg105 might be a unique binding site of CYP3A29 for T-2 toxin. These findings provide a possible interpretation for the structure-function relationship of CYP3A29 in the hydroxylation of T-2 toxin, giving a deeper understanding of the metabolic processes of T-2 toxin by cytochrome P450s. Since the hydroxylation and hydrolysis of T-2 toxin by CYP3A29 are detoxification reactions, CYP3A29 could be used as a detoxification enzyme. Moreover, our study indicates a protein engineering direction for this detoxification enzyme in the future, which may improve the efficiency of the prevention of T-2 toxin mediated hazard.

## Supporting Information

Figure S1 Amino acid sequence alignment of porcine CYP3A29, CYP3A22, CYP3A46 and human CYP3A4. Helices (red) are indicated by letters, and β-sheets (blue) are indicated by numbers above the sequences. The substrate recognition sites (SRSs) are indicated by pane (pink). Mutated sites are marked with fresh green.
(TIF)

Figure S2 Immunoblot analysis of recombinant CYP3A29 and its mutants. Microsomal proteins from Sf9 cells expressing R105A, R106A, F108A, S119A, K212A, F213A, F215A, R372A and E374A (from lanes 1 to 9) were subjected to SDS-PAGE. After electrophoresis, the proteins were transferred to a polyvinylidene fluoride membrane and probed with anti-human CYP3A4 immunoglobulin as described in Materials and Methods. Arrow indicates CYP3A29 and its mutants. M, protein molecular mass standard.
(TIF)

Figure S3 Nifedipine oxidation kinetics of recombinant CYP3A29 and its mutants. Nifedipine at concentration of 0, 2, 4, 8, 1, 20, 40, 60 and 80 µM were respectively incubated with 25 pmol CYP3A29 or its mutants at 37°C for 10 min as described in Materials and Methods. The Hill equation (v = V_max_ [S]^n^/(K_m_+[S]^n^)) was fitted by the data points. The solid red lines through the experimental data showed the best fits for the non-linear regression analysis using the Hill equation for sigmoidal kinetics. The standard deviations of three replicates did not exceed 10% of the mean values.
(TIF)

Figure S4 Accurate extracted ion chromatograms of the metabolites of T-2 toxin after incubation with recombinant CYP3A29 or its mutants. The CYP3A29 mutants include R105A, R106A, F108A, S119A, K212A, F213A, F215A, R372A and E374A.
(DOC)
