# Supplementary figures and images for: Structure-Function Analysis of Porcine Cytochrome P450 3A29 in the Hydroxylation of T-2 Toxin as Revealed by Docking and Mutagenesis Studies
Source: PLoS One. 2014 Sep 3;9(9):e106769. doi: 10.1371/journal.pone.0106769 (PMC4153680; doi:10.1371/journal.pone.0106769)

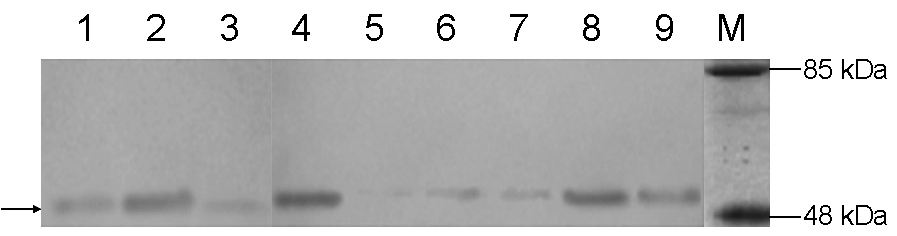

Supplement: Figure S2 — Immunoblot analysis of recombinant CYP3A29 and its mutants. Microsomal proteins from Sf9 cells expressing R105A, R106A, F108A, S119A, K212A, F213A, F215A, R372A and E374A (from lanes 1 to 9) were subjected to SDS-PAGE. After electrophoresis, the proteins were transferred to a polyvinylidene fluoride membrane and probed with anti-human CYP3A4 immunoglobulin as described in Materials and Methods. Arrow indicates CYP3A29 and its mutants. M, protein molecular mass standard. (TIF) [file pone.0106769.s002.tif]

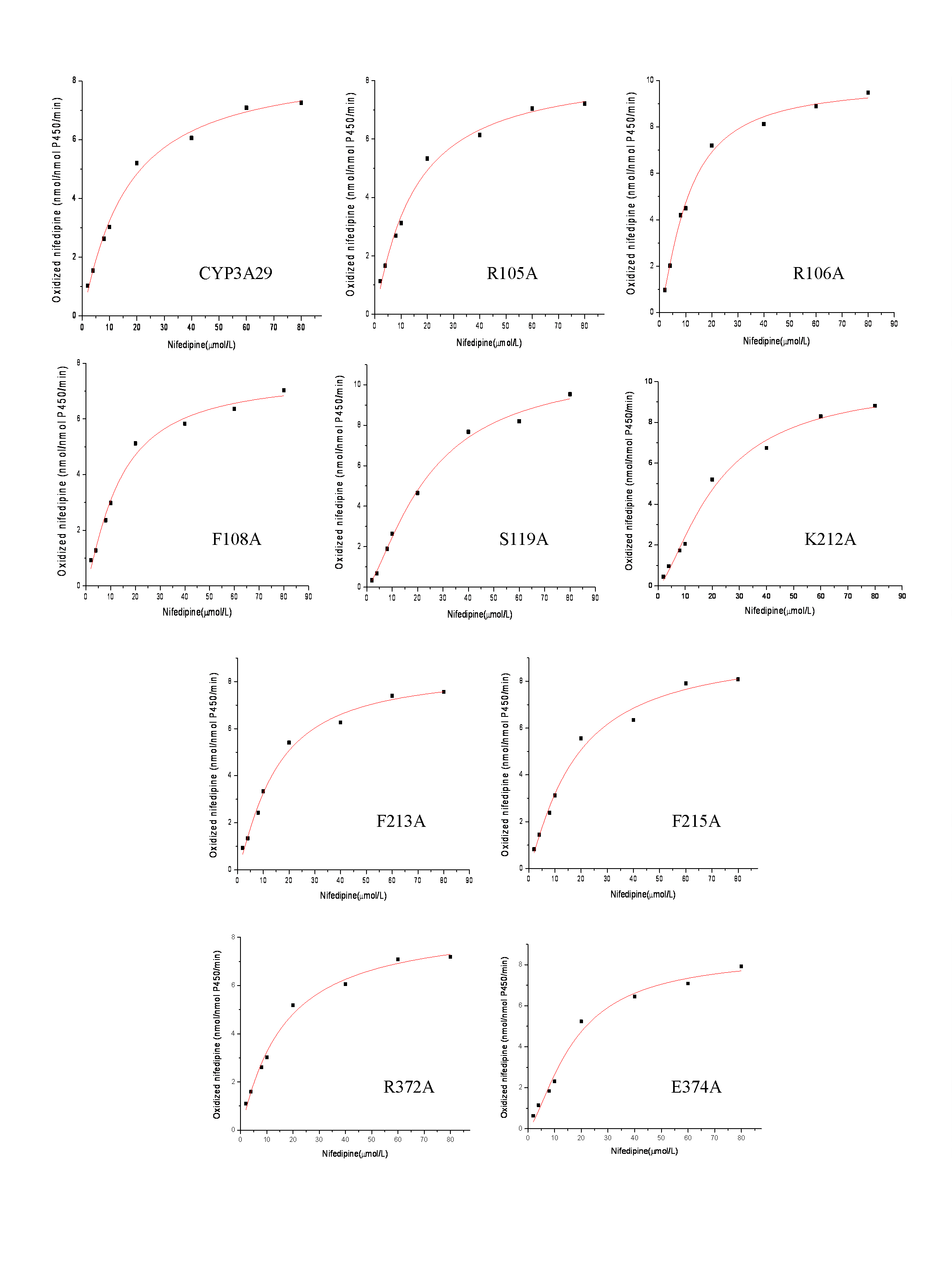

Supplement: Figure S3 — Nifedipine oxidation kinetics of recombinant CYP3A29 and its mutants. Nifedipine at concentration of 0, 2, 4, 8, 1, 20, 40, 60 and 80 µM were respectively incubated with 25 pmol CYP3A29 or its mutants at 37°C for 10 min as described in Materials and Methods. The Hill equation (v = Vmax [S]n/(Km+[S]n)) was fitted by the data points. The solid red lines through the experimental data showed the best fits for the non-linear regression analysis using the Hill equation for sigmoidal kinetics. The standard deviations of three replicates did not exceed 10% of the mean values. (TIF) [file pone.0106769.s003.tif]
