# Supplementary material for: Structure-Function Analysis of Porcine Cytochrome P450 3A29 in the Hydroxylation of T-2 Toxin as Revealed by Docking and Mutagenesis Studies
Source: PLoS One. 2014 Sep 3;9(9):e106769. doi: 10.1371/journal.pone.0106769 (PMC4153680; doi:10.1371/journal.pone.0106769)
Supplement: Figure S4 — Accurate extracted ion chromatograms of the metabolites of T-2 toxin after incubation with recombinant CYP3A29 or its mutants. The CYP3A29 mutants include R105A, R106A, F108A, S119A, K212A, F213A, F215A, R372A and E374A. (DOC) [file pone.0106769.s004.doc]

**CYP3A29**

3’-OH-T-2

NEO

**R105A**

**R106A**

**F108A**

**S119A**

**K212A**

**F213A**

**F215A**

**R372A**

**E374A**
